# The Stress-Response Gene *redd1* Regulates Dorsoventral Patterning by Antagonizing Wnt/β-catenin Activity in Zebrafish

**DOI:** 10.1371/journal.pone.0052674

**Published:** 2012-12-26

**Authors:** Qiang Feng, Xia Zou, Ling Lu, Yun Li, Yunzhang Liu, Jianfeng Zhou, Cunming Duan

**Affiliations:** 1 Key Laboratory of Marine Drugs (Ocean University of China), Chinese Ministry of Education, School of Medicine and Pharmacy, Ocean University of China, Qingdao, Shandong, China; 2 Department of Molecular, Cellular, and Developmental Biology, University of Michigan, Ann Arbor, Michigan, United States of America; Northwestern University Feinberg School of Medicine, United States of America

## Abstract

*REDD1/redd1* is a stress-response gene that is induced under various stressful conditions such as hypoxia, DNA damage, and energy stress. The increased REDD1 inhibits mTOR signaling and cell growth. Here we report an unexpected role of Redd1 in regulating dorsoventral patterning in zebrafish embryos and the underlying mechanisms. Zebrafish *redd1* mRNA is maternally deposited. Although it is ubiquitously detected in many adult tissues, its expression is highly tissue-specific and dynamic during early development. Hypoxia and heat shock strongly induce *redd1* expression in zebrafish embryos. Knockdown of Redd1 using two independent morpholinos results in dorsalized embryos and this effect can be rescued by injecting *redd1* mRNA. Forced expression of Redd1 ventralizes embryos. Co-expression of Redd1 with Wnt3a or a constitutively active form of β-catenin suggests that Redd1 alters dorsoventral patterning by antagonizing the Wnt/β-catenin signaling pathway. These findings have unraveled a novel role of Redd1 in early development by antagonizing Wnt/β-catenin signaling.

## Introduction

The Wnt/β-catenin signaling pathway, also known as the canonical Wnt signaling pathway, plays a pivotal role in embryogenesis and in adult tissue homeostasis [1]–[3]. Aberrant regulation of the Wnt/β-catenin pathway is also associated with many human diseases, such as cancer, osteoporosis, aging, and degenerative disorders [2], [4]. The transcriptional co-activator β-catenin is a key regulation step in this pathway. In the absence of Wnt ligands, cytoplasmic β-catenin is phosphorylated by the “destruction complex” consisting of Axin, APC, CK1 and GSK3β, resulting in β-catenin recognition by β-Trcp and subsequent degradation [5]. When Wnt ligands bind to the receptors Frizzled and co-receptor low-density lipoprotein receptor-related proteins 5 and 6 (LRP5/6), the Axin complex is recruited to the receptors and β-catenin phosphorylation and degradation are inhibited [5]. The stabilized β-catenin accumulates and translocates into the nucleus to form complexes with the transcription factors TCF/LEF and activates target gene expression.

In vertebrates, Wnt/β-catenin signaling plays a crucial role in dorsal organizer formation in early embryogenesis [6]–[8] and regulates anterior-posterior patterning at later stages [9], [10]. In zebrafish embryos, it has been reported that maternal and zygotic Wnt/β-catenin manifests different effects [11]. Maternal β-catenin localizes to the nucleus of dorsal marginal cells [12] and establishes dorsal cell fates before gastrulation [13]. Zygotic Wnt/β-catenin signaling in ventrolateral regions is required to initiate ventral cell fates after gastrulation [14], [15]. The nuclear localization of maternal β-catenin in the dorsal marginal cells leads to the expression of genes required for dorsal organizer formation, such as *bozozok (boz)*, *chordin (chd)*, and *goosecoid (gsc)* [16]–[18]. Loss of maternal β-catenin inhibits dorsal organizer formation. *Ichabod* mutants, in which maternal β-catenin 2 is absent, fail to form a normal embryonic shield [8], [13].

*REDD1* (*Regulated in Development and DNA damage responses 1*), also known as *RTP801/DDIT4/Dig2*, is a stress-response gene [19]. It was initially identified as a transcriptional target of p53 following DNA damage [20]. Subsequent studies suggest that it is also a hypoxia-inducible gene and regulated by HIF-1 [21], [22]. In addition to DNA damage and hypoxia, *REDD1* is up-regulated in response to energy stress [23], [24], food deprivation [25], glucocorticoid treatment [26], ER stress [27], [28], and high cell density [29]. The increased REDD1 inhibits mTOR signaling through the TSC1/TSC2 tumor-suppressor complex and inhibits cell growth [22], [23]. *REDD1* knockout mice are more tolerant to cigarette smoke-induced lung injury and emphysema, partly *via* increased mTOR signaling [30]. Scylla and Charybdis, two homologs of REDD1 in *Drosophila*, are also hypoxia induced. Flies that have lost both genes are more susceptible to hypoxia and mild overgrowth [31].

The goal of this study was to characterize the *redd1* gene, examine its expression and physiological regulation, and study its role in stress response *in vivo* using zebrafish as an experimental model. We found that zebrafish *redd1* is maternally deposited and has a highly tissue-specific and dynamic expression pattern in early embryogenesis. Loss- and gain-of-function studies suggest that Redd1 has a previously unrecognized role in regulating dorsoventral patterning by antagonizing Wnt/β-catenin signaling in zebrafish embryos.

## Results

### Zebrafish *redd1* Encodes a Conserved Protein and is Expressed in Many Tissues

**Figure 1:**
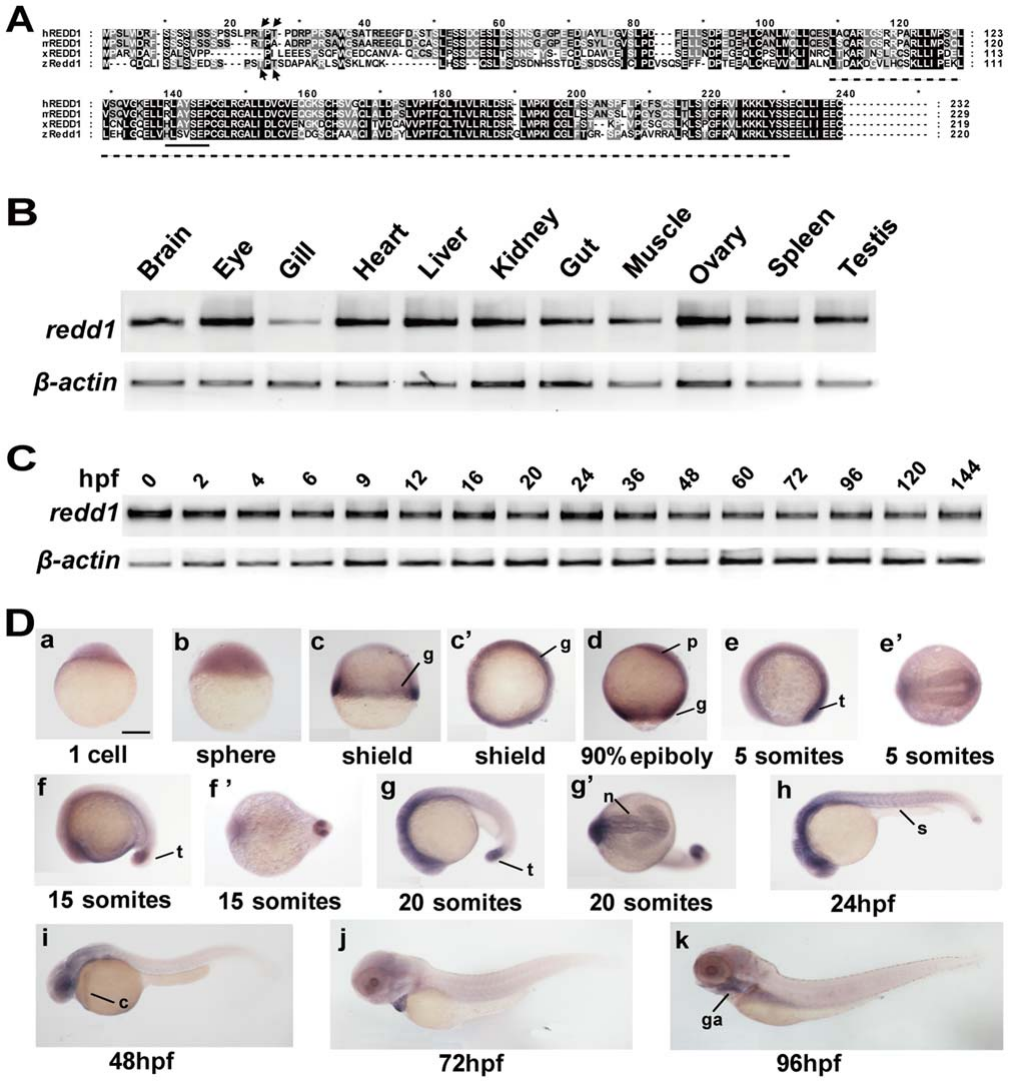
Zebrafish *redd1* encodes a conserved protein and is expressed in many tissues. A) Alignment of REDD1/Redd1 sequence from human, mouse, *Xenopus*, and zebrafish. Conserved residues are shaded. The RTP801_C domain is marked by a dotted line. Arrows mark the two Thr residues critical for human REDD1 phosphorylation and degradation. The conserved 14-3-3 binding site is indicated by a solid line. B) RT-PCR analysis of the indicated adult tissues. C) RT-PCR analysis of zebrafish embryos at the indicated stages. hpf, hours post fertilization. D) Whole mount *in situ* hybridization analysis of zebrafish embryos at the indicated stages. (a–c, d) Lateral views with the animal pole oriented at the top; (c′) Top view from the animal pole. (e, f, g, h–k) Lateral views with the anterior oriented toward the left. (e′, f′, g′) Ventral views with the anterior oriented toward the left. c, common cardinal vein; g, germ ring; ga, gill arches; n, neural ectoderm; p, prechordal plate/mesoderm; s, somite; t, tail bud. Scale bar = 200 µm.

By searching public databases and performing 5′- and 3′- rapid amplification of cDNA ends (RACE) experiments, we identified and cloned zebrafish *redd1* gene (GenBank accession number: HM114348). Like mammalian and amphibian REDD1, zebrafish Redd1 has a predicted RTP801_C domain (Fig. 1A) and this domain shares a sequence identity of 61%, 60% and 61% to that of human, mouse, and frog, respectively. There is a conserved 14-3-3 binding site in the middle region (Fig. 1A). Recent studies in human REDD1 revealed that REDD1 undergoes GSK3β-dependent phosphorylation through Thr23 and Thr25, and this leads to its degradation [32]. These two residues are conserved in zebrafish Redd1 at positions 18 and 20 (Fig. 1A). These structural features suggest that this is indeed a bona fide Redd1. This conclusion is further supported by phylogenetic, genome structure, and synteny analyses, showing that zebrafish *redd1* is a human *REDD1* ortholog (Fig. S1).

In the adult stage, *redd1* mRNA was detected by RT-PCR in all tissues examined, albeit at lower levels in gills (Fig. 1B). RT-PCR analysis showed that *redd1* mRNA was detectable throughout early development, ranging from fertilized eggs to 6 days old larvae (Fig. 1C). Whole mount *in situ* hybridization analysis indicated that r*edd1* mRNA was detected in fertilized eggs (one-cell stage) and in all blastodermal cells at the sphere stage (Fig. 1D, panels a and b). At the shield stage, *redd1* mRNA expression became restricted to the germ ring, where mesodermal precursors reside (Fig. 1D, panels c and c′). During gastrulation and segmentation stages, *redd1* mRNA was abundant in the prechordal plate/mesoderm (Fig. 1D, panel d), tail bud (Fig. 1D, panels e-g′), and the neural ectoderm (Fig. 1D, panels e-g′). At 24 hpf and thereafter, *redd1* mRNA was expressed mainly in the neural ectoderm, somites (Fig. 1D, panel h), common cardinal vein (Fig. 1D, panels i and j), and the gill arches (Fig. 1D, panel k). These results indicate that *redd1* mRNA is maternally deposited. Although it is ubiquitously expressed in many adult tissues, *redd1* expression during early development is highly tissue-specific and dynamic.

### Zebrafish *redd1* is Up-regulated Under Multiple Stressors

**Figure 2:**
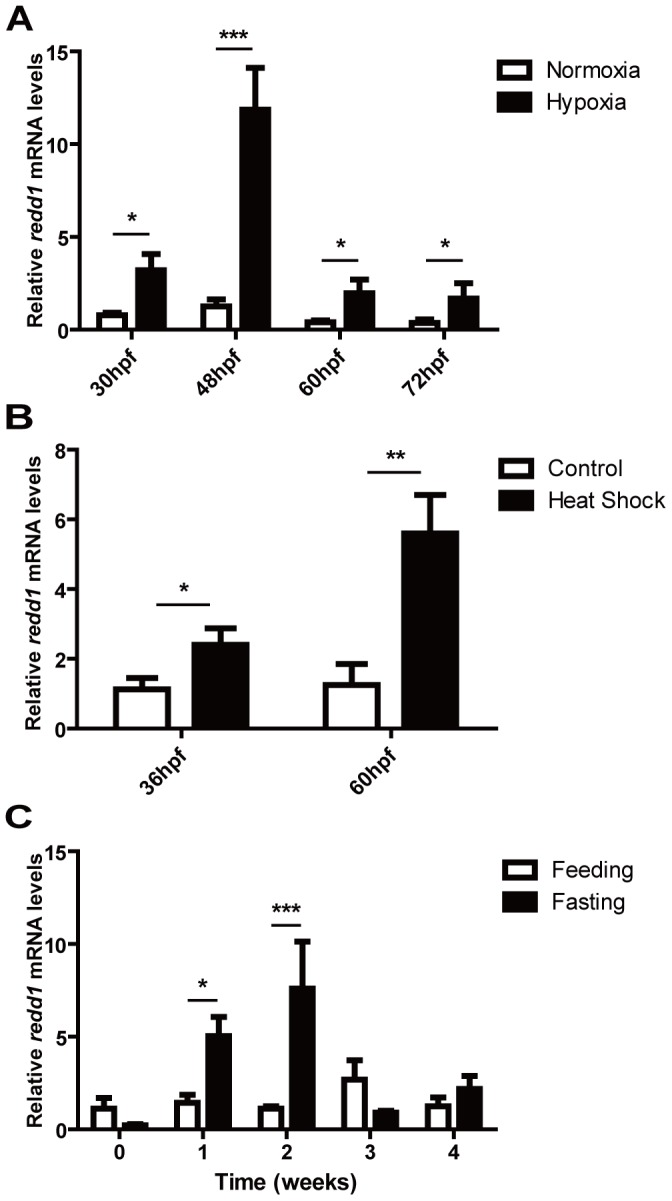
Zebrafish *redd1* is a stress-response gene. A) Effect of hypoxia. 6, 24, 36, and 48 hpf old embryos were subjected to 24 h hypoxia treatment (10% of ambient O_2_ levels). Total RNA was isolated at the indicated developmental stages. The levels of *redd1* mRNA were measured by qRT-PCR and normalized by the *β-actin* mRNA levels. In this and all subsequent figures, the mRNA levels are expressed as a relative value of the control group. Values are means ± S.E. (n = 3). * *P*<0.05, ** *P*<0.01, and *** *P*<0.001. B) Effect of heat shock. Embryos were subjected to 1 h heat shock (37°C) treatment in every 12 h and sampled at 36 hpf and 60 hpf. The levels of *redd1* mRNA were measured as described above. C) Effect of food deprivation. Total RNA was isolated from juvenile fish with constant feeding (Feeding) or fasting (Fasting) at indicated time points. The levels of *redd1* mRNA were measured and presented as described above.

The effects of hypoxia, heat shock, and food deprivation on the expression of *redd1* were studied by quantitative real-time RT-PCR. Embryos of 6, 24, 36 and 48 hours post fertilization (hpf) were subjected to physical hypoxia treatment for 24 hours. In all these stages, *redd1* mRNA levels were up-regulated under hypoxic conditions. The *redd1* mRNA levels increased to nearly five folds (P<0.05) at 30 hpf, ten folds (P<0.001) at 48 hpf, and remained elevated at 60 and 72 hpf (Fig. 2A). The *redd1* mRNA levels were also increased to approximately three folds (P<0.05) at 36 hpf, and five folds at 60 hpf (P<0.01) by heat shock treatments (Fig. 2B). We also studied the effect of food deprivation in juvenile and adult fish. The expression levels of *redd1* were significantly increased (P<0.05) when fasted for one week, and reached their zenith (P<0.001) when fasted for two weeks. The expression then decreased to the control group levels in week three and week four (Fig. 2C), suggesting this is a stress response. Similar induction of *redd1* expression by food deprivation was seen in one-year old adult fish (data not shown). Collectively, these results showed that zebrafish *redd1* is a stress-response gene in all life stages from early embryos to adults.

### *Redd1* Knockdown Results in Dorsalized Embryos

**Figure 3:**
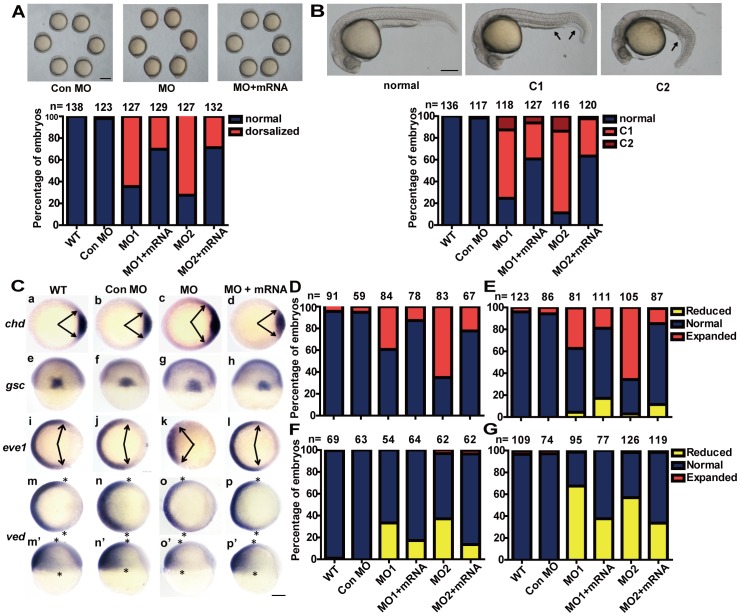
Knockdown of *redd1* results in dorsalized embryos. A) Effects of *redd1* knockdown. Upper panels are representative views of zebrafish embryos (at 5 somite stage) injected with control MO, *redd1* targeting MO (MO1 or MO2), or *redd1* targeting MO+ *redd1* mRNA (MO+mRNA). Lateral views with anterior up. Scale bar = 200 µm. The results are from three independent experiments and the total embryo number is given at the top. B) Effects of *redd1* knockdown. The experimental groups are the same as in A). Representative images of 24 hpf zebrafish embryos are shown in the upper panel. Scale bar = 200 µm. The percentage of dorsalized embryos in each group is shown in the lower panel. The total number of embryos is shown on the top of each column. C–G) Effects of *redd1* knockdown on the expression of dorsoventral marker genes. Embryos described in A) and B) were analyzed by *in situ* hybridization using the indicated probes. Representative images are shown in C). Panels a–d and i–p are animal pole views with dorsal to the right; panels e–h are dorsal views with animal pole up; panels m′–p′ are lateral views with dorsal to the right and animal pole up. Arrows indicate the edges of the *chd* and *eve1* mRNA expression domains. Asterisks indicate the edges of the *ved* mRNA expression domain. Scale bar = 200 µm. The percentage of embryos in each category is shown in D (*chd*), E (*gsc*), F (*eve1*), and G (*ved*). The results are from three independent experiments and the total embryo number is given at the top.

As mentioned above, the *redd1* transcript is maternally deposited and is expressed in a dynamic and tissue-specific manner in early development. To investigate the possible role of endogenous *redd1* in embryonic development, morpholino-mediated knockdown of *redd1* was carried out. The efficacies of these *redd1* MOs were verified by co-injection with a *redd1* 5′-UTR-GFP expression construct (Fig. S2). Approximately 70–80% of embryos injected with *redd1* MOs exhibited mild dorsalized phenotypes, *i.e*., a protruding tailbud that does not extend around the yolk as far as in the wild type, but lies in a more vegetal position (Fig. 3A). At 24 hpf, the dorsalized phenotype were more obvious and can be morphologically classified into two groups: C1 and C2, according to a previous study [33]. Embryos in the C1 group exhibited partial loss of the caudal ventral fin, a bent tail, and shortened yolk sac extension (Fig. 3B, arrows). The majority of dorsalized embryos after MO1 or MO2 injection belonged to this group (Fig. 3B and Fig. S3). Embryos in the C2 group showed nearly complete loss of caudal ventral fins, a severely bent tail, and shortened yolk sac extension (Fig. 3B, arrows). A much lower proportion of embryos injected with MO1 or MO2 belonged to this group (Fig. 3B). The *redd1* MO-induced dorsalization was partially neutralized by co-injection with *redd1* mRNA (70% *vs.* 30% at the 5–10 somite stage, and 80% *vs.* 40% at 24 hpf), suggesting that the MO-induced morphological changes were indeed due to the loss of Redd1.

To determine whether the observed phenotypes were due to changes in dorsoventral pattern formation, whole mount *in situ* hybridization analysis was performed using several dorsal-ventral axis marker genes. *chordin* (*chd)* and *goosecoid* (*gsc)* expression marks the dorsal axial mesoderm at the shield stage [17], [18]. The expression of *even-skipped-1* (*eve1*) and *ventral edema* (*ved*), on the other hand, is restricted in the ventral non-axial mesoderm [34], [35]. Knockdown of *redd1* with either MO1 or MO2 resulted in an expansion of the *chd* and *gsc* expression domains (Fig. 3C, panels c and g, Fig. 3D and 3E). Co-injection with *redd1* mRNA reduced the *chd* and *gsc* expression to the wild type and control MO group levels (Fig. 3C, panels d and h, Fig. 3D and 3E). In contrast, embryos with *redd1* knockdown showed reduced *eve1* and *ved* expression (Fig. 3C, panels k, o and o′, Fig. 3F and 3G). Co-injection with *redd1* mRNA restored *eve1* and *ved* expression to the wild type and control MO levels (Fig. 3C, panels i, p and p′, Fig. 3F and 3G). These results suggest that *redd1* knockdown causes dorsalization in zebrafish embryos.

### Redd1 Ventralizes Embryos by Inhibiting Wnt/β-catenin Signaling

**Figure 4:**
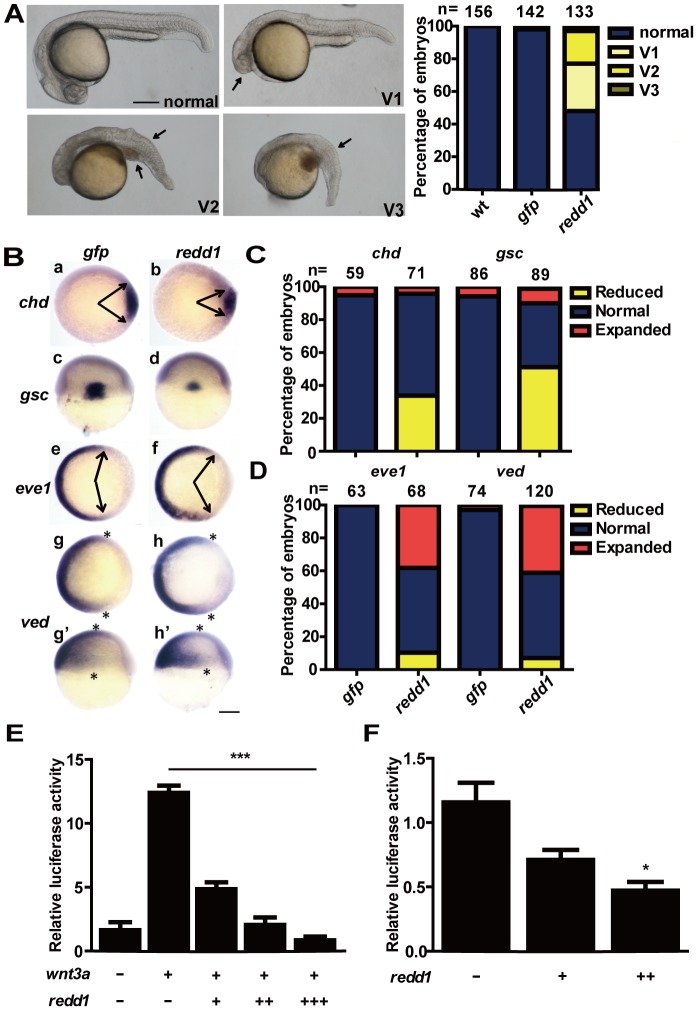
Redd1 ventralizes embryos and inhibits Wnt signaling. A) Left panel: classification of phenotypes embryos caused by forced expression of Redd1. 1–2 cell stage embryos were injected with *redd1* mRNA. They were raised to 24 hpf and examined. The percentage of embryos in each category is shown in the right panel. B–D) Effects of Redd1 expression. Embryos described in A) were analyzed by whole mount *in situ* hybridization using the indicated probes. Representative images are shown in B). Panels a–b and e–h are animal pole views with dorsal to the right; panels c–d are dorsal views with animal pole up; panels g′–h′ are lateral views with dorsal to the right and animal pole up. Arrows indicate the edges of the *chd* and *eve1* mRNA expression domains. Asterisks indicate the edges of the *ved* mRNA expression domain. Scale bar = 200 µm. The percentage of embryos in each category is calculated and shown in C) (*chd* and *gsc*) and D) (*eve1* and *ved*). The results are from three independent experiments and the total embryo number is given at the top. E) Redd1 inhibits Wnt3a activity. One-cell stage embryos were injected with 20 pg *wnt3a* mRNA alone or co-injected with 10 pg (+), 50 pg (++), or 100 pg (+++) *redd1* mRNA. TCF/LEF-luciferase reporter DNA was injected in all groups. The embryos were raised to the shield stage and the luciferase activity was determined. Values are means ± S.E. (n = 3). *** *P*<0.001. F) Redd1 inhibits endogenous Wnt signaling. Embryos were injected with TCF/LEF-luciferase reporter DNA without and with 10 pg (+) or 100 pg (++) *redd1* mRNA. The embryos were raised to the shield stage and the luciferase activity was measured. Values are means ± S.E. (n = 3). * *P*<0.05 compared to the TCF/LEF control group.

We next investigated the effect of Redd1 forced expression. Redd1 was GFP tagged and its expression was confirmed by Western blotting analysis (Fig. S4). Injection with 250 pg *redd1* mRNA resulted in ventralized phenotype in ∼50% embryos. The ventralized embryos were morphologically classified into three groups: V1, V2, and V3 (Fig. 4A). Embryos in the V1 group showed smaller eyes and reduced head (Fig. 4A, arrow). Embryos in the V2 group showed no eye, severely reduced head and notochord, and expanded posterior somites. Embryos in the V3 group completely lacked the head, notochord, and had expanded posterior somites (Fig. 4A, arrows). The effects of *redd1* forced expression on the dorsoventral marker genes were examined next. Embryos injected with *redd1* mRNA resulted in reduced *chd* and *gsc* expressions (Fig. 4B, panels b and d and Fig. 4C), and elevated *eve1* and *ved* expressions (Fig. 4B, panels f, h and h′ and Fig. 4D). These results indicate that forced expression of Redd1 ventralizes zebrafish embryos.

Since the ventralized phenotypes caused by Redd1 expression resembled much of those seen in the *ichabod* and *bozozok* (*boz*), two mutants lacking maternal *β-catenin-2* [8], [13] and its target gene *boz* [36], we postulated that Redd1 may act by affecting the Wnt/β-catenin signaling pathway. To test this hypothesis, we investigated whether Redd1 inhibits Wnt/β-catenin signaling using a Wnt signal-responsive reporter, Topflash, which contains tandem repeats of the TCF/LEF response element [37]. Co-injection of *wnt3a* mRNA with Topflash plasmid DNA resulted in a strong induction in the Topflash reporter activity (Fig. 4E). This induction was significantly and dose-dependently inhibited by co-injection with several doses of *redd1* mRNA (Fig. 4E). Forced expression of Redd1 also inhibited endogenous Wnt signaling activity, as indicated by reduced Topflash reporter activity in *redd1* mRNA-injected embryos (Fig. 4F). A similar inhibition of Wnt3a activity by Redd1 was observed when tested in the human embryonic kidney (HEK) 293T cells *in vitro* (Fig. S5). These data indicate that Redd1 negatively regulates Wnt signaling.

**Figure 5:**
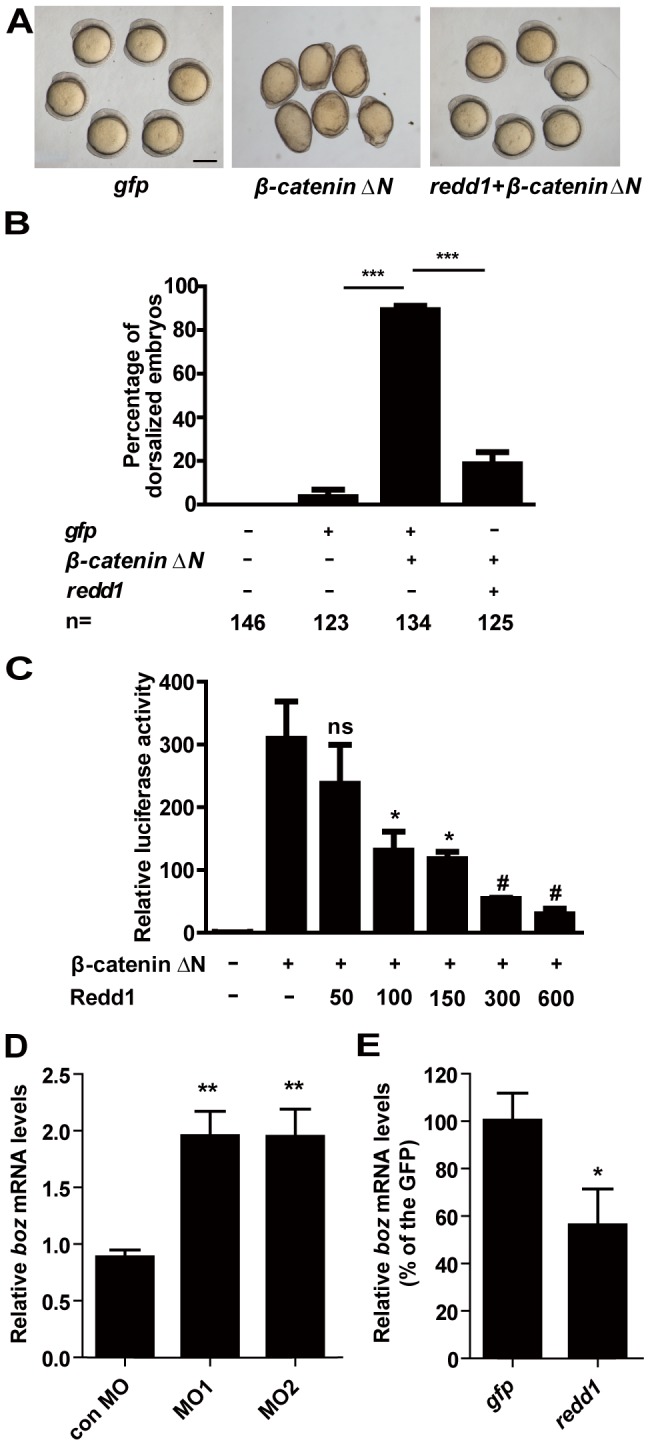
Redd1 inhibits β-catenin action. A and B) Redd1 inhibits β-catenin action *in vivo*. Representative view of *gfp* mRNA-, *β-catenin ΔN* mRNA-, and *β-catenin ΔN* mRNA *+ redd1* mRNA-injected embryos at 5 somite stage is shown in A). Scale bar = 200 µm. Quantitative results are shown in B). The results are from three independent experiments and the total embryo number is given at the bottom. *** *P*<0.001. C) Redd1 inhibits β-catenin activity *in vitro*. HEK293T cells were transfected with β-catenin ΔN plasmid DNA and increasing doses of Redd1 plasmid DNA, together with the same amount of TCF/LEF-luciferase reporter DNA. Cells transfected with TCF/LEF-luciferase reporter DNA alone were used as negative control (−).Values are means ± S.E., n = 3. ns, not significant, * and #, *P*<0.05 and *P*<0.001 compared to the β-catenin ΔN group. D) *Redd1* knockdown increases *boz* expression. Embryos were injected with control MO, *redd1* targeting MO1 or MO2 at one-cell stage. The embryos were raised to the dome stage. The *boz* mRNA levels were measured by quantitative real-time RT-PCR. E) Forced expression of Redd1 decreases *boz* expression. Embryos were injected with *gfp* mRNA or *redd1* mRNA at one-cell stage and were analyzed at dome stage. The *boz* mRNA levels were measured by quantitative real-time RT-PCR.

We used a constitutively active β-catenin ΔN mutant which lacking the first 45 residues in the N-terminal region [38] to further investigate the role of Redd1 in regulating Wnt/β-catenin signaling. Injection of *β-catenin ΔN* mRNA resulted in dorsalized phenotype in more than 80% injected embryos (Fig. 5A, 5B). When the same amount of *β-catenin ΔN* mRNA was co-injected with *redd1* mRNA, the percentage of dorsalized embryos was reduced to 20% (Fig. 5A, 5B). A similar effect was also found in HEK293T cells (Fig. 5C), suggesting that Redd1 antagonizes β-catenin activity both *in vivo* and *in vitro*.

We examined the effects of *redd1* knockdown and forced expression on the expression of *boz*, a direct target gene of maternal β-catenin, at the dome stage (4.3 hpf) when zygotic Wnt/β-catenin is not yet functional [39], [40]. Compared with the control group, embryos injected with *redd1* MO1 or MO2 had significantly higher *boz* mRNA levels (Fig. 5D). Furthermore, forced expression of Redd1 significantly decreased *boz* mRNA levels (Fig. 5E), indicating that Redd1 inhibits maternal β-catenin activity.

## Discussion

In this study, we have shown that zebrafish *redd1* is a syntenic gene of human REDD1. Sequence comparison suggests that Redd1 is structurally conserved. Taking advantage of the zebrafish model, we have mapped the spatial and temporal expression pattern of *redd1* during early development and made several new findings. First, zebrafish *redd1* is a maternal gene. Both RT-PCR and *in situ* hybridization can easily detect *redd1* mRNA in fertilized eggs. Second, zebrafish *redd1* mRNA is expressed throughout early development. Intriguingly, its expression in early embryos is highly tissue-specific. Zebrafish *redd1* mRNA is highly expressed in the germ ring at the shield stage. Later on, it is abundantly expressed in the prechordal plate/mesoderm, tail bud, and the neural ectoderm. At 24 hpf and thereafter, *redd1* mRNA was expressed mainly in the neural ectoderm, somites, cardinal vein, and the gill arches. This tissue-specific expression pattern in zebrafish embryos is consistent with what have been reported in *Drosophila* and rodents. The *Drosophila* REDD1/Redd1 homologs, *scylla* and *charybde*, are abundantly expressed in the dorsal domain during gastrulation and later in the central nervous system and cardiac precursor cells [41]. In developing mouse embryos, *REDD1* is expressed in the apical ectodermal ridge, a cluster of primitive ectoderm that is critical for induction of limb development; in ectodermally derived tissues such as the whisker pad and eyelid; as well as the developing cartilage of the limbs, tail, and cranium [20]. In adult zebrafish, *redd1* mRNA was detectable in all adult tissues examined, although its level in the gill was lower. The ubiquitous expression pattern fits its function as a stress-response gene. In human and mice, *REDD1* is ubiquitously expressed at low levels [42], [43]. These results suggest that the temporal and spatial expression of *REDD1*/*redd1* is more dynamic than previously thought. Although it is ubiquitously expressed in many adult tissues, its expression during early development is highly tissue-specific and dynamic.

Mammalian *REDD1* has been shown to be induced by hypoxia [21], [22], [42], energy stress [23], [24], and food deprivation [25]. When rats were subjected to food deprivation for 18 hours, both *Redd1* mRNA and protein levels increase dramatically. A subsequent 45 min re-feeding reduced them back to the baseline levels [25]. In zebrafish, *redd1* mRNA was also up-regulated by food deprivation. Furthermore, we found that *redd1* is strongly induced in early embryos by hypoxia and heat shock, suggesting a possible role of *redd1* as a stress-response gene during embryogenesis. Indeed, our loss-of-function and gain-of-function analyses suggest that Redd1 plays a critical role in regulating dorsoventral patterning in zebrafish. *Redd1* morphants had a protruding tailbud and a loss of the caudal ventral fin, which are seen in dorsalized zebrafish mutants [44], [45]. Molecular analysis using various dorsal and ventral marker genes revealed an expansion of expression domains of the dorsal marker genes *chd* and *gsc* and a reduction in ventral marker genes such as *eve1* and *ved*. Moreover, forced expression of Redd1 caused a reduction in dorsoanterior structures and an expansion of ventroposterior structures.

How does a stress-response gene in the mTOR signaling pathway regulate dorsoventral patterning in embryogenesis? In this study, we provided several lines of evidence supporting the notion that Redd1 regulates dorsoventral patterning by antagonizing Wnt/β-catenin signaling. We showed that co-expression of Redd1 and Wnt3a in zebrafish embryos significantly inhibited Wnt3a activity. Likewise, Redd1 expression inhibited the β-catenin ΔN-induced dorsalizing effect *in vivo* and its activity *in vitro*. Importantly, *redd1* knockdown significantly increased and Redd1 overexpress reduced the expression of *boz*, a target gene of maternal β-catenin. These results strongly argue that Redd1 antagonizes maternal β-catenin signals. Recently, it was shown that CHOP (GADD153), which belongs to the same protein family as Redd1, binds to TCFs, thereby inhibiting Wnt/TCF activity [46]. Whether Redd1 inhibit Wnt/β-catenin signaling using a similar mechanism needs further investigation. The fact that Redd1 antagonizes Wnt/β-catenin signaling, however is in agreement with the expression patterns of these genes. In early zebrafish embryos, *redd1* is mainly expressed at the germ ring, prechordal mesoderm, tail bud, and anterior neuroectoderm. These expression sites are similar to those of *wnt8* [47], *β-catenin* [13], and *tcf3* [48], . Therefore, *redd1* has overlapping expression domains with the major components of the Wnt/β-catenin signaling pathway, suggesting that they may interact with each other *in vivo*.

In conclusion, our study in zebrafish suggests that the stress-response gene *redd1* has a previously unrecognized developmental role. Redd1 regulates dorsoventral patterning by inhibiting Wnt/β-catenin signaling. These findings add new knowledge on the regulation of dorsoventral patterning in early development. Recent studies have suggested that mammalian REDD1 regulates cell growth *via* the mTOR signaling pathway [21], [24], [25]. Interestingly, Wnt can activate mTOR signals through GSK3 phosphorylation of TSC2, although this activation does not involve β-catenin-dependent transcription [50]. Thus, Wnt signals may regulate transcription through β-catenin as well as translation through mTOR *via* two different signaling branches downstream of GSK3. Since *REDD1*/*redd1* is induced by DNA damage, hypoxia, and starvation and because it both down regulates mTOR signaling and antagonizes Wnt/β-catenin signaling, it may act as a key player in these processes under different conditions. Aberrant regulation of the Wnt/β-catenin signaling is associated with many human diseases [3]. Mutations that result in up-regulation of nuclear β-catenin levels are often linked to increased tumorigenesis [5]. Similarly, down-regulation of REDD1 expression was observed in a number of human cancers [22]. It will be of interest to investigate β-catenin signaling in those tumor tissues and determine its relationship to reduced REDD1 activity.

## Materials and Methods

### Chemicals and Reagents

M-MLV Reverse Transcriptase, Riboprobe® System—T3/T7, and the Dual-Glo™ Luciferase Assay System were purchased from Promega (Madison, WI, USA). KOD plus DNA polymerase was purchased from TOYOBO (Shanghai, China). iQ SYBR Green Supermix was purchased from Bio-Rad (Hercules, CA, USA). mMESSAGE mMACHINE mRNA synthesis kit was purchased from Ambion (Austin, TX, USA). DIG-UTP and Anti-Digoxigenin-AP were purchased from Roche (Indianapolis, IN, USA). Morpholino oligonucleotides were purchased from Gene Tools, LLC (Philomath, OR, USA). Anti-GFP antibody (TP401) was purchased from Torrey Pines Biolabs (Houston, TX, USA). Dulbecco's Modified Eagle's Medium (DMEM) and OPTI-MEM I reduced serum medium were purchased from GIBCO, Invitrogen (Carlsbad, CA, USA). Lipofectamine 2000 transfection agent was purchased from Invitrogen (Carlsbad, CA, USA).

### Experimental Animals

Fish were kept at very low densities and the water was replaced at regular intervals with biological filters and UV sterilization to keep it clean and specific pathogen free. The containers were overflowed and made of plastic. Wild-type zebrafish (*Danio rerio*) were maintained on a 14 h/10 h light/dark cycle at 28°C and fed twice daily with *Artemia nauplia*. Embryos were obtained by natural cross. Fertilized eggs were raised at 28.5°C in the embryo-rearing solution and staged according to Kimmel *et al.* [51]. 2-Phenylthiourea [0.003% (w/v)] was added to the embryo-rearing solution in some experiments to inhibit embryonic pigment formation. All surgery was performed under tricaine for anesthesia of fish, and all efforts were made to minimize suffering. All experiments were conducted in accordance with the guidelines established by the University Committee on the Use and Care of Animals at Ocean University of Chinaand the ARRIVE (Animal Research: Reporting of *In Vivo* Experiments) guidelines [52]. The protocol was approved by the Committee on the Ethics of Animal Experiments, Ocean University of China (Permit Number: 09001).

### Molecular Cloning and Sequence Analysis

Using human REDD1 (NP_061931) amino acid sequence as a query, we searched the zebrafish genome database (http://asia.ensembl.org/Danio_rerio/blastview) by TBLASTN and found a REDD1-like sequence. The full-length complementary DNA (cDNA) was determined by 5′- and 3′- RACE using the SMART RACE cDNA amplification kit (Clontech, Mountain View, CA, USA) following the manufacturer's instruction.

The sequence alignment, phylogenetic and synteny analyses were performed as described previously [53]. Drosophila *scylla* and *charybde* genes were used as outgroups. Synteny analysis was carried out based on the zebrafish (*Danio rerio* Zv9) and the human (*Homo sapiens* GRCh37) genome databases.

### RT-PCR and Whole Mount *in situ* Hybridization

Total RNA was isolated from embryos and adult zebrafish tissues using TRIzol reagent (TaKaRa). One microgram of total RNA was reverse transcribed to single-strand cDNA using M-MLV reverse transcriptase according to the manufacturer's instructions with oligo(dT)_18_ (Sangon, Shanghai, China) as first-strand primers. Quantitative real-time RT-PCR was carried out in an iCycler iQ Multicolor real-time PCR detection system (Bio-Rad Laboratories) using iQ SYBR Green Supermix. The primers for RT-PCR were: *redd1* (forward, 5′- ATGCAAGATCAGTTGATTTCCAGCC-3′; reverse, 5′-TCAGCATTCTTCAATCAGGAGCTCT-3′); *β-actin* (forward, 5′-CTTGCGGTATCCACGAGAC-3′; reverse, 5′-GCGCCATACAGAGCAGAA-3′). The primers for quantitative real-time RT-PCR were: *redd1* (forward, 5′-TGGACTCTGACTCCGACAACC-3′; reverse, 5′-ACCACTTCTTTACACAACGCCTC-3′); *boz* (forward, 5′- GATGTACTGCTGCTGCGTTCC-3′; reverse, 5′-CTGCTCCGTCTGGTTGTCG-3′); *β-actin* (forward, 5′-ACAGGGAAAAGATGACACAG-3′; reverse, 5′-AGAGTCCATCACGATACCAG-3′). Each sample was measured in duplicate. *redd1* and *boz* mRNA levels were calculated using 2^−ΔΔCt^ method [54] and presented as relative (fold) levels normalized to the level of *β-actin*.

For whole-mount *in situ* hybridization analysis, plasmids containing complete CDS were linearized by restriction enzyme digestion, followed by *in vitro* transcription reactions with either T3 or T7 RNA polymerase, to generate antisense or sense riboprobes using DIG RNA labeling mix. The specificity of the riboprobes was verified by dot-blot assay, and they did not cross-react with each other's target. Hybridization was carried out as described previously [55].

### Physiological Regulation of *redd1* Gene Expression

For the hypoxia experiment, 20–30 embryos at 6 hours post fertilization (hpf), 24 hpf, 36 hpf, and 48 hpf were subjected to physical hypoxia (10% of ambient O_2_ levels, oxygen was depleted by bubbling water with nitrogen gas) for 24 h. The dissolved oxygen content in the hypoxia group was 0.7±0.06 mg/L for embryos, whereas the normal ambient oxygen concentration was 6.9±0.5 mg/L. In the heat shock experiment, 20–30 embryos were subjected to 1 h 37°C heat shock treatment in every 12 h and sampled at 36 hpf and 60 hpf. To examine the effect of food deprivation, two-month old juvenile fish were fasted for four weeks. At each week, four fish were randomly chosen from the fasting and feeding control groups respectively. RNA was isolated and subjected to reverse transcription and quantitative real-time RT-PCR.

### Plasmid Construction

For functional analysis, cDNA encoding the zebrafish *redd1* open reading frame (ORF) (with the stop codon deleted) was amplified by PCR using KOD plus DNA polymerase. The PCR product was subcloned into the pCS2+EGFP expression vector and verified by DNA sequencing.

### Capped mRNA Synthesis, Morpholinos, Microinjection, and Western Immunoblot

Capped mRNA synthesis and microinjection were performed essentially as previously reported [55]. Antisense Morpholinos targeting zebrafish *redd1* had the sequences MO1: 5′-CAAGCCGTGTGTATCCTCAAGTCTG-3′ and MO2: 5′-TGGTGAAATAGTCCGTAACAAAGAC-3′. A standard control morpholino (5′-CCTCTTACCTCAGTTACAATTTATA-3′) was injected as a negative control. Western immunoblot was performed as described previously [56].

### Cell Culture and Luciferase Reporter Assay

Human embryonic kidney (HEK) 293T cells and HeLa cells were maintained in DMEM supplied with 10% fetal bovine serum. Cells were seeded into 12-well plates to reach 60–70% confluence at the time of transfection. Plasmids were transfected in duplicate with Lipofectamine 2000. Luciferase activities were measured 24 h after the transfection using a Dual-Luciferase assay kit. 500 ng Topflash DNA, 100 ng *Renilla* DNA, and pCS2+ plasmid was used to adjust the DNA amount to1.5 µg/well. Topflash luciferase activity was normalized to that of *Renilla* luciferase activity. The *in vivo* luciferase assay was performed as reported by Sun *et al*. [57]. Briefly, the plasmids and mRNAs were mixed prior to injection. The topflash plasmid was mixed with the Renilla plasmid in a ratio of 100 pg : 20 pg. 1 nl dose was injected into 1- to 2-cell-stage zebrafish embryos. At shield stage, a group of 20–30 embryos for each sample was grinded and lysed in 50 µl 1× passive lysis buffer (Promega) at room temperature. After low centrifugation, the supernatant was used for assays using the Dual-Luciferase assay kit (Promega) following the manufacturer's instruction.

### Statistical Analysis

Values are presented as means ± S.E. Differences among groups were analyzed by one-way ANOVA followed by Tukey's Multiple Comparison Test or by t-Test using GraphPad Prism version 5.01 (San Diego, CA, USA). Significance was accepted at *P*<0.05.

## Supporting Information

Figure S1 **Zebrafish** ***redd1*** **is orthologous to human** ***REDD1*** **.** A) Phylogenetic tree of vertebrate REDD1. The tree was built using the Neighbor-Joining + JTT matrix-based method. Phylogenetic analyses were conducted in MEGA4. Drosophila *charybde* and *scylla* genes were used as outgroups. Similar results were obtained using the Maximum Likelihood method. B) Comparison of human, mouse, and zebrafish *REDD1/redd1* gene structure. Exons are shown as boxes (protein coding region in filled box and UTR in open box). Introns are shown as lines. Analysis was obtained from the Blat program at UCSC Genome Browser (http://genome.ucsc.edu) C) Zebrafish *redd1* is syntenic to human *REDD1*. Genes are represented by lines. Transcriptional direction is indicated by arrow. Zebrafish *redd1* is located on chromosome 12 and human *REDD1* is located on chromosome 10. Gene order was obtained from the Ensembl Genome Browser (http://www.ensembl.org).
(TIF)

Figure S2 **The efficacy of** ***redd1*** **MOs.** A GFP reporter was constructed containing the entire 5′-UTR and partial ORF of *redd1*. Embryos were injected with the GFP reporter DNA, reporter DNA + Control MO, reporter DNA + *redd1* targeting MO1, or reporter DNA + *redd1* targeting MO2. The injected embryos were raised to tail bud stage and photographed under a fluorescence microscope.
(TIF)

Figure S3 **Phenotypes of zebrafish** ***redd1*** **MO injected embryos.** Embryos were injected with *redd1* targeting MO1 or MO2 at 1-cell stage and raised to 24 hpf. Lateral views are shown with the anterior oriented toward the left. The percentage of embryos with the indicated phenotype and the total number of embryos examined are shown in the right corner. Scale bar = 200 µm.
(TIF)

Figure S4 **Western immunoblot analysis of wild type (WT),** ***gfp*** **(GFP) mRNA-, and** ***redd1-gfp*** **(Redd1) mRNA-injected embryos.** Injected embryos were raised to 6–7 hpf and subjected to SDS-PAGE followed by immunoblot analysis using a GFP antibody.
(TIF)

Figure S5 **Redd1 inhibits Wnt3a activity** ***in vitro*** **.** HEK293T cells were transfected with Wnt3a plasmid DNA and two doses (300 ng and 600 ng) of Redd1 plasmid DNA, together with TCF/LEF-luciferase reporter DNA. Cells transfected with TCF/LEF-luciferase reporter DNA alone were used as negative control. Values are means ± S.E., n = 3. ***, *P*<0.001 compared to the Wnt3a group.
(TIF)
