# Supplementary figures and images for: The Stress-Response Gene redd1 Regulates Dorsoventral Patterning by Antagonizing Wnt/β-catenin Activity in Zebrafish
Source: PLoS One. 2012 Dec 26;7(12):e52674. doi: 10.1371/journal.pone.0052674 (PMC3530439; doi:10.1371/journal.pone.0052674)

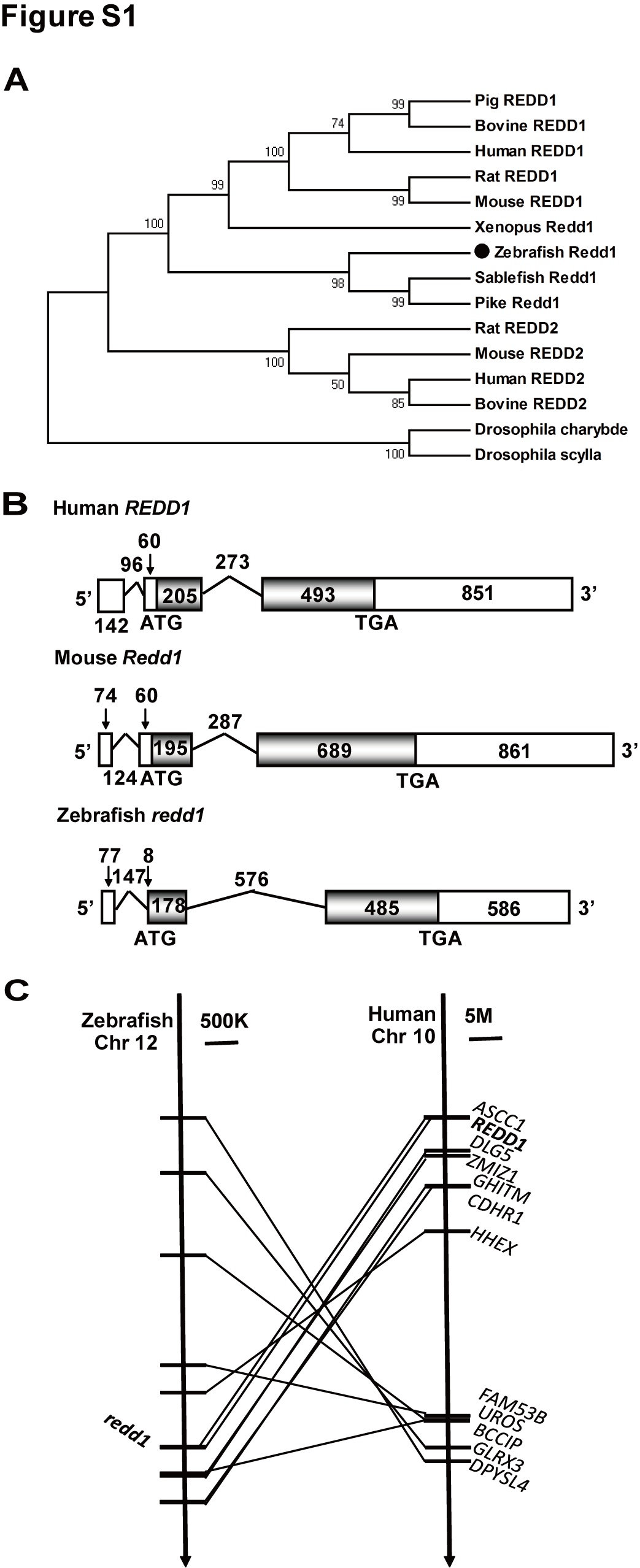

Supplement: Figure S1 — Zebrafish redd1 is orthologous to human REDD1 . A) Phylogenetic tree of vertebrate REDD1. The tree was built using the Neighbor-Joining + JTT matrix-based method. Phylogenetic analyses were conducted in MEGA4. Drosophila charybde and scylla genes were used as outgroups. Similar results were obtained using the Maximum Likelihood method. B) Comparison of human, mouse, and zebrafish REDD1/redd1 gene structure. Exons are shown as boxes (protein coding region in filled box and UTR in open box). Introns are shown as lines. Analysis was obtained from the Blat program at UCSC Genome Browser (http://genome.ucsc.edu) C) Zebrafish redd1 is syntenic to human REDD1. Genes are represented by lines. Transcriptional direction is indicated by arrow. Zebrafish redd1 is located on chromosome 12 and human REDD1 is located on chromosome 10. Gene order was obtained from the Ensembl Genome Browser (http://www.ensembl.org). (TIF) [file pone.0052674.s001.tif]

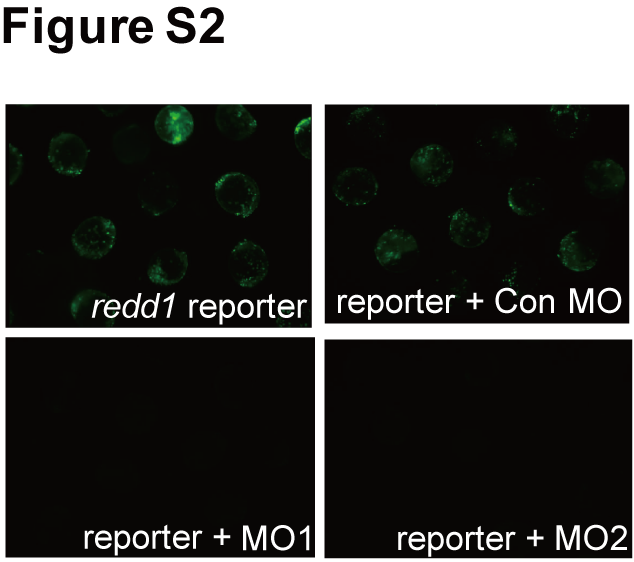

Supplement: Figure S2 — The efficacy of redd1 MOs. A GFP reporter was constructed containing the entire 5′-UTR and partial ORF of redd1. Embryos were injected with the GFP reporter DNA, reporter DNA + Control MO, reporter DNA + redd1 targeting MO1, or reporter DNA + redd1 targeting MO2. The injected embryos were raised to tail bud stage and photographed under a fluorescence microscope. (TIF) [file pone.0052674.s002.tif]

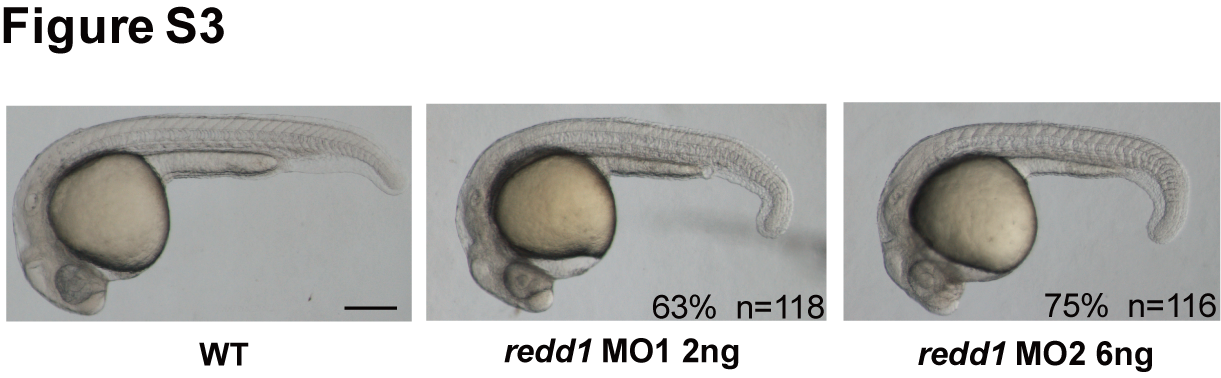

Supplement: Figure S3 — Phenotypes of zebrafish redd1 MO injected embryos. Embryos were injected with redd1 targeting MO1 or MO2 at 1-cell stage and raised to 24 hpf. Lateral views are shown with the anterior oriented toward the left. The percentage of embryos with the indicated phenotype and the total number of embryos examined are shown in the right corner. Scale bar = 200 µm. (TIF) [file pone.0052674.s003.tif]

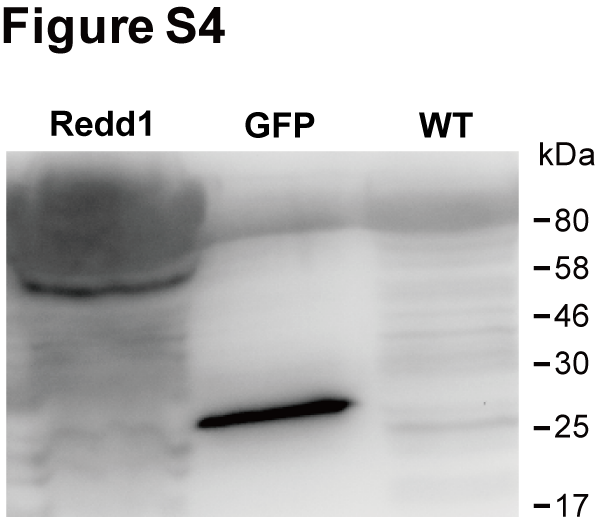

Supplement: Figure S4 — Western immunoblot analysis of wild type (WT), gfp (GFP) mRNA-, and redd1-gfp (Redd1) mRNA-injected embryos. Injected embryos were raised to 6–7 hpf and subjected to SDS-PAGE followed by immunoblot analysis using a GFP antibody. (TIF) [file pone.0052674.s004.tif]

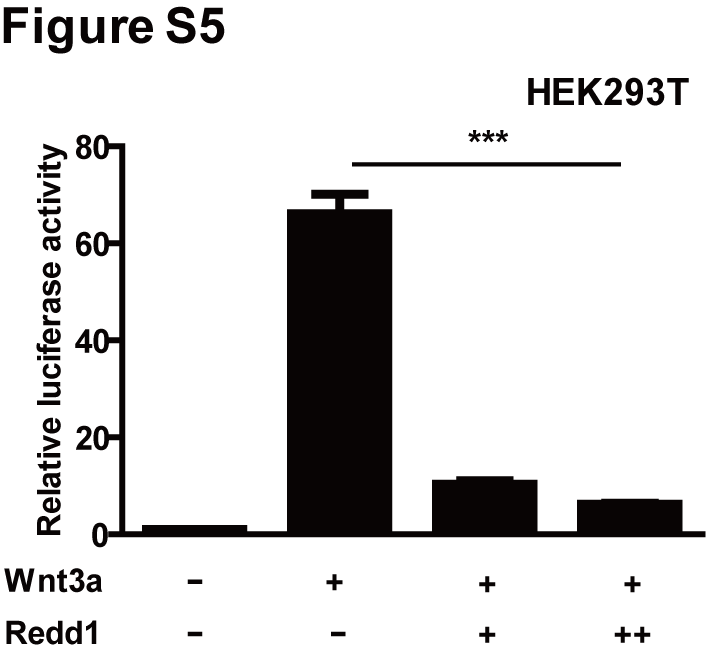

Supplement: Figure S5 — Redd1 inhibits Wnt3a activity in vitro . HEK293T cells were transfected with Wnt3a plasmid DNA and two doses (300 ng and 600 ng) of Redd1 plasmid DNA, together with TCF/LEF-luciferase reporter DNA. Cells transfected with TCF/LEF-luciferase reporter DNA alone were used as negative control. Values are means ± S.E., n = 3. ***, P<0.001 compared to the Wnt3a group. (TIF) [file pone.0052674.s005.tif]
